# Investigating genetic, antigenic, and structural diversity in the *Neisseria gonorrhoeae* outer membrane protein, PorB: implications for vaccine design

**DOI:** 10.1128/mbio.01309-25

**Published:** 2025-08-25

**Authors:** Odile B. Harrison, Margaret Bash, Fidel Ramirez-Bencomo, Angela Thistlethwaite, Rebekah Jones, Lenka Stejskal, Eduard Sanders, Ian M. Feavers, Ann Jerse, Jeremy P. Derrick, Christoph M. Tang, Martin C. J. Maiden

**Affiliations:** 1 Nuffield Department of Population Health, University of Oxford, Oxford, United Kingdom; 2 U.S. Food and Drug Administration, Washington, DC, USA; 3 School of Biological Sciences, Lydia Beck Institute of Immunology and Inflammation, The University of Manchester, Manchester, United Kingdom; 4 Sir William Dunn School of Pathology, University of Oxford, Oxford, United Kingdom; 5 The Aurum Institute, Johannesburg, South Africa; 6 Department of Biology, University of Oxford, Oxford, United Kingdom; 7 Department of Microbiology and Immunology, Uniformed Services University 1685 , Bethesda, Maryland, USA; GSK Vaccines, Siena, Italy

**Keywords:** gonorrhea, vaccine, genomics, machine-learning

## Abstract

Vaccines targeting *Neisseria gonorrhoeae* are needed to reduce disease burden and help address the problem of antimicrobial resistance, with an understanding of relationships between gonococcal genetics and molecules influencing diversity, infection, and the immune response essential for developing effective vaccine formulations. Whole-genome sequence data can be used to investigate these relationships among thousands of gonococcal isolates, allowing the study of antigenic diversity on a population scale. Such analyses typically examine antigenic diversity occurring in complete protein sequences, generating mean diversity indices and phylogenetic analyses that can inform on vaccine potential; however, to detect and measure the immune responses elicited, epitope characterization within an antigen helps guide vaccine formulations, with epitopes commonly located in surface-exposed regions of a protein. Here, we analyzed the genetic diversity of the major gonococcal antigen, PorB, in WGS from 22,227 *N*. *gonorrhoeae* isolates. We characterized the diversity of all eight surface-exposed outer membrane loops, or variable regions (VRs), and generated a PorB VR subtyping scheme to facilitate the global and temporal detection of circulating PorB subtypes. These analyses identified the presence of dominant VR combinations that persisted over time, indicative of (i) epistatic interactions between VRs and (ii) positive selection. Strain-specific, anti-PorB IgG responses directed toward distinct VR subtypes were detected in sera obtained from participants vaccinated with 4CMenB. The deconstruction of PorB into each surface-exposed loop provides a powerful approach for evaluating vaccine candidates: the methods used here allow immunodominant regions to be detected, which is invaluable for further vaccine investigations.

**IMPORTANCE:**

In the context of rising global gonorrhea cases, the development of vaccines becomes a priority; however, *N. gonorrhoeae* antigenic diversity and its ability to evade the immune system complicate vaccine development. This study characterizes the genetic diversity of the outer membrane protein, PorB, a key component of the outer membrane and a major gonococcal antigen. Using genomics and machine-learning techniques, this research identified dominant PorB variants that drive the immune response, proposing potential vaccine candidates and improving our understanding of the evolutionary forces maintaining genome structure and biological fitness. Understanding these processes is crucial for designing vaccines that effectively target *N. gonorrhoeae* and combat the spread of multidrug-resistant gonococci.

## INTRODUCTION

The bacterium, *Neisseria gonorrhoeae* (the gonococcus), causes the sexually transmitted infection gonorrhea. Rising cases of gonorrhea, combined with a rapid increase in multidrug resistance, have resulted in this infection becoming a major public health concern. As a result, vaccines targeting *N. gonorrhoeae* are a priority; however, vaccine development has been hampered by the extensive antigenic diversity exhibited by *N. gonorrhoeae* and its ability to subvert the host immune response (1). Antigenic variation is a strategy used by many pathogens to avoid host immunity and proliferate. In *N. gonorrhoeae,* this is achieved through multiple mechanisms, including (i) antigen on/off switching, known as phase variation (e.g., the opacity proteins and lipo-oligosaccharide biosynthesis LOS) (2, 3), (ii) horizontal gene transfer (HGT) and recombination of immunogenic surface-exposed regions using RecA-dependent processes (e.g., *pilE*) (4), and (iii) amino acid variation in surface-exposed epitopes, resulting in the expression of novel antigenic variants (e.g., *tbpB*) (5). Effective vaccine formulations should ideally include conserved, surface-exposed, constitutively expressed antigenic determinants and, over 30 highly, that is, 80% sequence identity, to moderately, that is, 50% sequence identity, conserved surface-exposed antigens have been identified in *N. gonorrhoeae* whole genome sequence data from several thousand isolates (5, 6).

Surface-exposed regions in antigens targeted by the immune response are subject to immune selection pressure, resulting in diversification (7). The high conservation observed in many gonococcal antigens suggests, however, that these may not be subject to strong immune selection and that antigenic variation may be achieved using mechanisms distinct from phase variation, given that many of these are also constitutively expressed. One such mechanism may include genetic reassortment, where extensive intraspecies HGT results in the re-distribution of allelic variants throughout the gonococcal population, a phenomenon consistent with the observation of a limited number of antigen alleles shared between gonococcal isolates (5, 6).

Genetic reassortment is a strategy used by many pathogens to evade the host immune response, including segmented RNA viruses such as influenza A, where co-infection and the exchange of genome segments generate reassortant viruses exhibiting extensive strain diversity (8). Genetic reassortment can therefore be evolutionarily advantageous as it increases population-level genetic diversity. It can also maintain genome structure through directional and/or stabilizing selection of recombination events, conferring a fitness advantage, with deleterious events negatively selected against. Thus, although some of the antigenic diversity observed in *N. gonorrhoeae* will be a consequence of genetic reassortment, evolutionary dynamics will select for beneficial antigen allelic combinations in association with genome content. This is evidenced through the observation of discrete gonococcal genome lineages that persist for decades and is consistent with linkage disequilibrium despite genome recombination (9). Exposing prevailing antigenic repertoires that are circulating in gonococcal populations and how these associate with genome structure will therefore be important in the choice of candidate antigens to be included in vaccine formulations.

Here, gonococcal genetic reassortment is examined in the outer membrane protein, PorB. Intraspecies reassortment of the surface-exposed outer membrane loops of PorB has been described and, along with its extensive diversity and immune-modulatory effects, PorB has not been considered to be an ideal vaccine candidate (5, 10). Presumably, the antigenic diversity present in PorB is a consequence of interactions between the gonococcus and the immune system; however, PorB acts primarily as a porin for nutrient acquisition and is thus essential for growth. The role PorB plays in gonococcal biology, therefore, dictates that reassortment may be positively selected for change or inversely, negatively selected against, to maintain functionality (11). We investigated this through machine-learning approaches and genomic data and individually characterized each surface-exposed outer membrane loop, also known as the variable region (VR). A total of 5,706 porB alleles from 22,227 *N*. *gonorrhoeae* isolates were analyzed and a PorB subtyping designation generated, allowing prevailing PorB subtypes circulating in the population to be characterized and examined in combination with genome lineage. Using protein microarray data containing diverse PorB peptides, we assessed PorB IgG responses in sera obtained from participants vaccinated with 4CMenB and sera obtained during and after infection (clinicaltrials.gov identification: NCT 04297436) and identified anti-PorB IgG responses directed toward distinct VRs. This multi-disciplinary approach evaluated the implications of this variation in vaccine design.

## RESULTS

### Data set selection and genomic analyses

Publicly available whole-genome sequence data for 22,227 isolates were retrieved from PubMLST (https://pubmlst.org). Isolates originated from all continents and dated from 1940 to 2022. Draft assemblies consisted of an average of 171 contigs with an average genome size of 2,154,457 bp. A total of 828 MLST STs were present, the most frequent being MLST ST-1901 (*n* = 2,322), followed by ST-9363 (*n* = 1,985) and ST-7363 (*n* = 1,491), with 402 STs represented by single isolates. A total of 397 LINcode lineages were identified (Tables S1). Kenyan gonococcal whole-genome sequence data (WGS) with matched sera belonged to 12 lineages with LINcode lineages 0_16_0 (*n* = 19) and 0_0_47 (*n* = 13) the most prevalent (*n* = 35) (Table S2).

### NEIS2020 (*porB*) genetic analyses

A total of 5,706 *porB* alleles were identified, of which alleles 537 (*n* = 779, 4%), 513 (*n* = 679, 3%), 517 (*n* = 522, 2%), and 544 (*n* = 450, 2%) were the most frequent. A total of 3,744 (17%) were unique, occurring once in the data set. Of the 5,706 *porB* alleles, 402 (7%) were *porB1a* and 5,304 (93%) *porB1b*. A total of 2,191 (10%) *N*. *gonorrhoeae* isolates possessed *porB1a* alleles, with the remaining 20,036 (90%) isolates possessing *porB1b*. Fifteen of the 47 Kenyan isolates with matched plasma samples possessed *porB1a* variants (32%), with the remaining 32/47 (68%) harboring *porB1b*.

**Fig 1:**
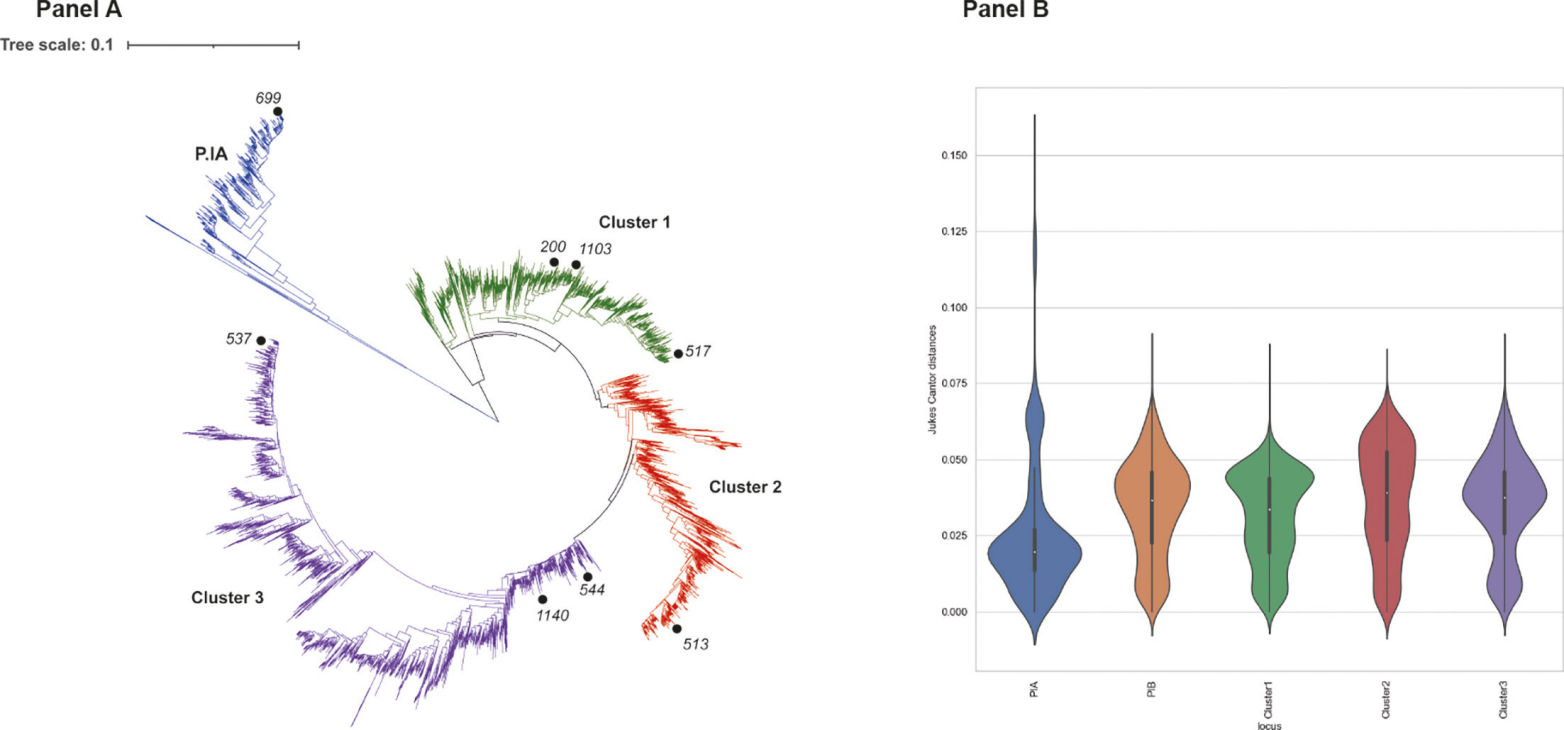
PorB phylogenetic analyses. (A) Neighbor-joining tree of PorB alleles. Alleles included in microarray analyses are indicated by black circles. The tree was generated from an amino acid alignment of 5,706 PorB alleles using the alignment tool, MUSCLE (12). Alignments were translated into their deduced amino acid sequences from which a neighbor-joining tree with 100 bootstraps was generated. The resulting tree was edited and annotated using iTOL (13). (B) Violin plots depicting the range of nucleotide diversity found. The Python package, Cogent3 (14), was employed to determine pairwise distances using the (15) (JC69) substitution model in all *porB* alleles (15). JC69 values were then visualized through violin plots generated using the seaborn Python package to allow the range of diversity present to be observed.

Phylogenetic analyses of deduced amino acid sequences from all 5,706 PorB alleles identified four large clusters: one consisting solely of P.IA alleles with the remaining three clusters comprised of P.IB (Fig. 1A). The P.IA cluster included 402 alleles found in 2,191 isolates, P.IB cluster 1 consisted of 1,727 alleles found in 6,301 isolates, P.IB cluster 2 included 1,220 alleles found in 4,079 isolates, and P.IB cluster 3 was comprised of 2,357 alleles harbored by 9,648 isolates. The Jukes-Cantor (1969) (JC69) substitution model was used to estimate nucleotide substitutions and the amount of diversity present. This model assumes that the nucleotide substitution rate is the same for all ATGC pairs and was chosen given the short branches observed in the neighbor-joining tree (Fig. 1A). Mean JC69 values varied from 0.024 in *porB1a* to 0.035 for *porB1b* (Table 1). Violin plots depicting the range of JC69 values present in each cluster identified deviation from the mean ranging from 0 to 0.160 for *porB1a* and 0 to 0.090 for *porB1b*, indicative of extensive diversity (Fig. 1B).

**TABLE 1:** PorB genetic diversity indices^*a*^

PorB variant	Number of nt alleles (aa)	Mean bp length (aa)	Number of identical nt sites	Number of identical aa sites	Pairwise nt sequence identity	Pairwise aa sequence identity	Mean JC69
PIA	402 (402)	984 (327)	699 (67.5%)	189 (55.4%)	97.4%	94.5%	0.024
PIB	5,304 (5,304)	1,045 (347)	105 (7%)	149 (36.4%)	91.7%	86.8%	0.034
Cluster 1	1,727 (1,727)	1,043 (347)	711 (56.3%)	197 (44.2%)	96.1%	93.4%	0.031
Cluster 2	1,220 (1,220)	1,048 (348)	757 (62.0%)	215 (51.1%)	95.8%	92.8%	0.037
Cluster 3	2,357 (2,357)	1,046 (348)	684 (50.8%)	184 (40.0%)	95.4%	94.6%	0.035

^*a*^ nt: nucleotide; aa: amino acid; JC69: Jukes Cantor 1969.

Eight PorB peptides had been chosen for inclusion in the microarray slide fabrication, based on their frequency in the gonococcal sample, with phylogenetic analyses confirming that these were representative of known PorB alleles, with alleles 200 (from FA1090), 517, and 1103 found in cluster 1, allele 513 in cluster 2, and alleles 537, 544, and 1140 in cluster 3. Allele 699 represented P.IA.

### PorB peptide loop characterization

Prior to sequence-based approaches, gonococci were typed using monoclonal antibodies targeting PorB, which provided a serological typing tool to delineate the gonococcus (16). After this, eight surface-exposed loops have been described, and these were annotated VR1 through to VR8 here. VR2, VR4, and VR8 were the most conserved, with 50, 62, and 98 peptide alleles found, respectively (Table 2). A total of 525 alleles were found for VR1, with 558 defined for VR3. VR5 exhibited the most diversity with 1,278 peptides identified in the 22,227 gonococcal isolates.

**TABLE 2:** VR family distributions in PorB clusters and across the data set^*a*^

VR (aa length)	Consensus sequence	No of alleles	Frequency in PorB alleles	Frequency in PIB cluster 1	Frequency in PIB cluster 2	Frequency in PIB cluster 3	Frequency in PIA
Loop 1							
VR1-1 (22)	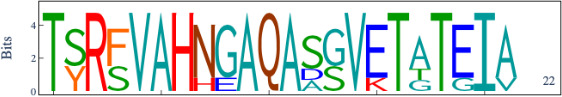	5	16	0	0	0	16
VR1-2 (24)	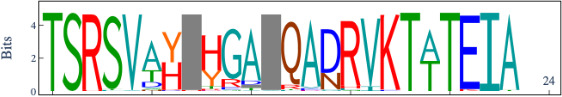	78	377	0	0	1	376
VR 1-3 (22)	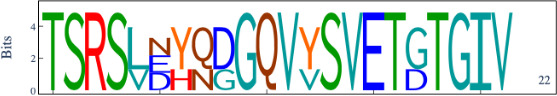	3	4	0	0	0	4
VR1-4 (24)	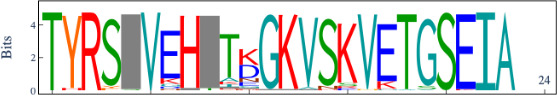	65	2,058	710	827	517	4
VR1-5 (25)	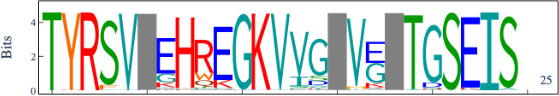	241	3,251	1,017	393	1,839	2
Loop 2							
VR2-1 (11)	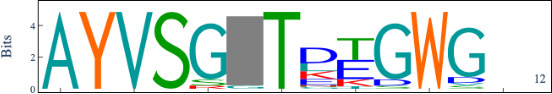	6	377	0	0	1	376
VR2-2 (11)	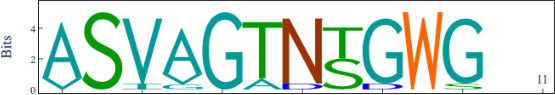	12	5,310	1,725	1,220	2,356	9
VR2-3 (11)	AYVSGTNTGWG	1	17	0	0	0	17
Loop 3							
VR3-1 (34)	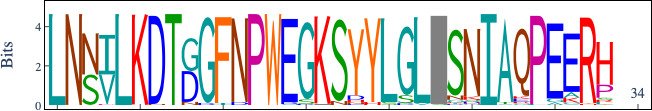	87	400	0	0	0	400
VR3-2 (44)	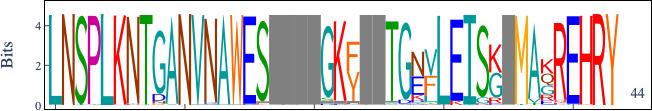	204	2,602	787	692	1,121	2
VR3-3 (37)	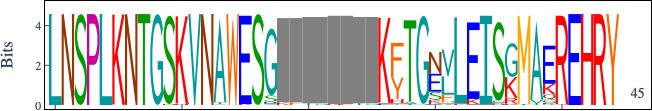	95	709	159	109	441	0
VR3-4 (37)	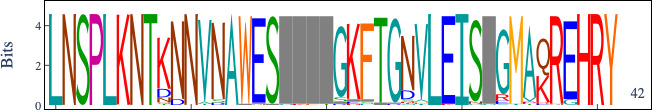	71	698	568	43	87	0
VR3-5 (37)	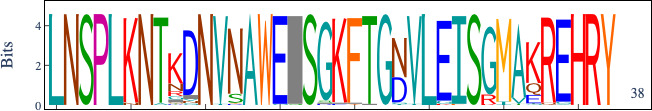	75	1,083	177	249	657	0
VR3-6 (37)	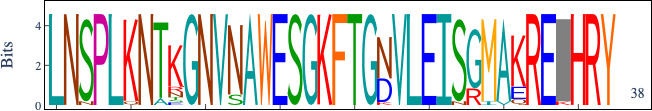	23	212	36	127	49	0
VR3-7 (37)	LNSPLKNTNGNVNAWESGKFTGNVLEISGMVKREHRY	1	2	0	0	2	0
Loop 4							
VR4-1 (11)	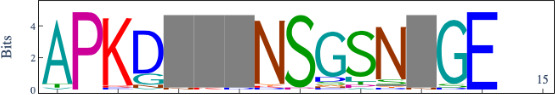	37	5,299	1,726	1,220	2,353	0
VR4-2 (12)	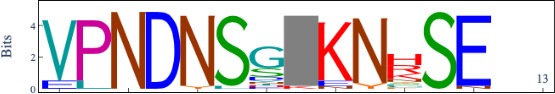	25	403	1	0	4	398
Loop 5							
VR5-1 (9)	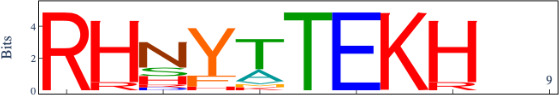	17	399	2	0	1	396
VR5-2 (26)	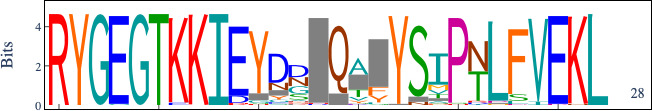	72	151	93	11	47	0
VR5-3 (23)	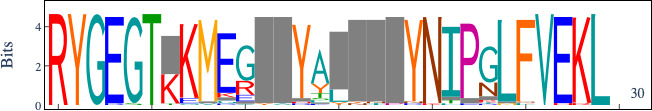	47	148	113	3	32	0
VR5-4 (25)	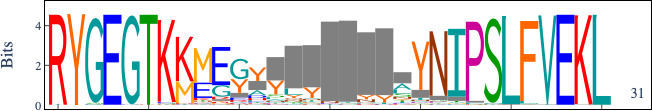	283	1,683	874	98	711	0
VR5-5 (26)	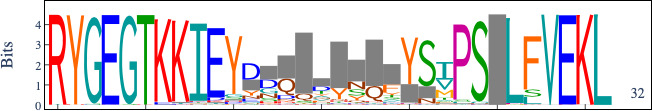	768	3,238	624	1,090	1,524	0
VR5-6 (22)	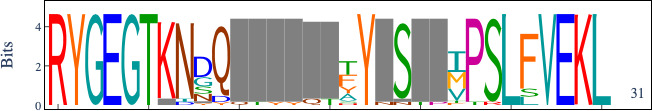	24	46	12	7	27	0
VR5-7 (26)	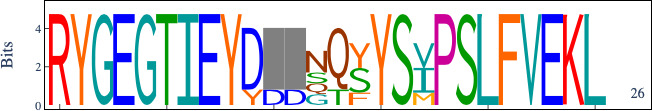	5	6	0	3	3	0
VR5-8 (26)	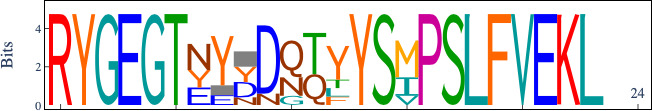	7	9	0	7	2	0
VR5-9 (26)	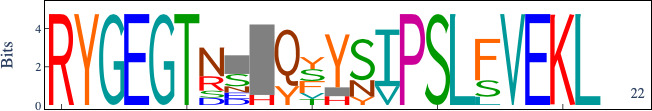	8	9	1	1	7	0
VR5-10 (26)	RYGEGTKSQYYSIPSLFVEKL	2	3	0	0	3	0
Loop 6							
VR6-1 (14)	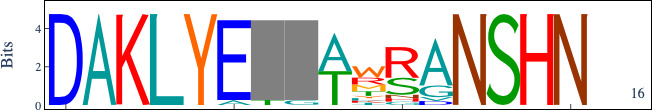	15	114	69	12	33	0
VR6-2 (14)	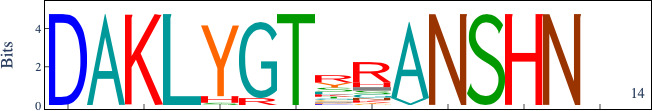	29	231	26	33	172	0
VR6-3 (14)	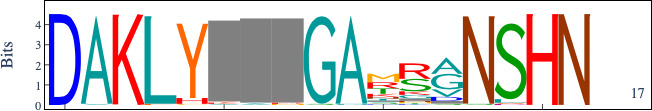	55	1,945	1,244	642	59	0
VR6-4 (13)	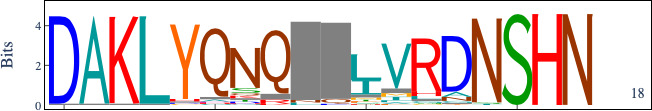	51	678	77	28	573	0
VR6-5 (14)	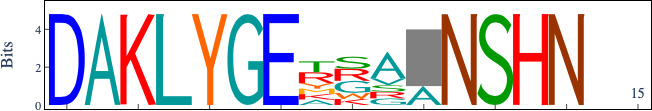	5	7	4	3	0	0
VR6-6 (14)	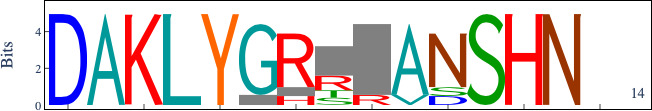	7	14	1	2	11	0
VR6-7 (13)	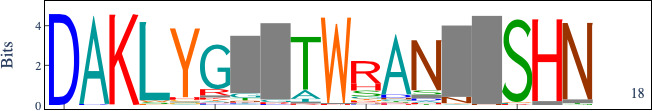	79	2,259	262	492	1,505	0
VR6-8 (14)	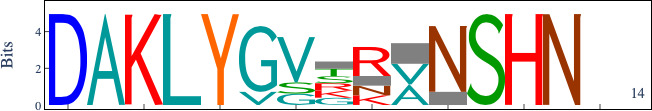	2	4	4	0	0	0
VR6-9 (14)	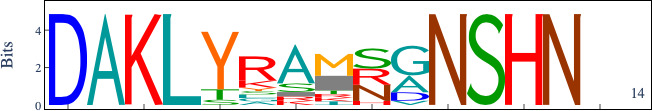	11	47	37	7	2	1
VR6-10 (14)	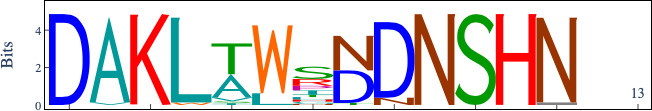	23	403	3	1	2	397
Loop 7							
VR7-1 (13)	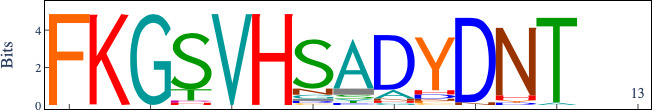	29	1,972	3	383	1,584	2
VR7-2 (13)	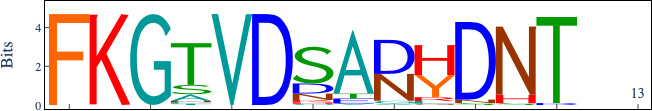	44	2,891	1,566	725	599	1
VR7-3 (13)	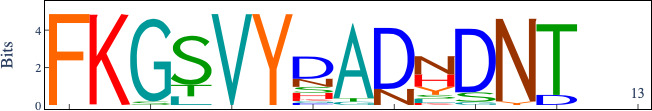	17	424	4	3	24	393
VR7-4 (13)	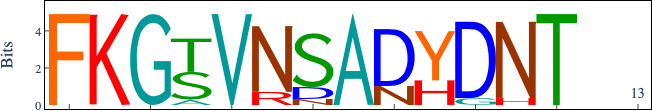	11	271	151	32	88	0
VR7-5 (13)	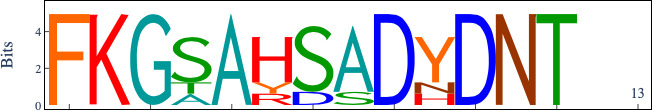	6	119	0	70	48	1
VR7-6 (13)	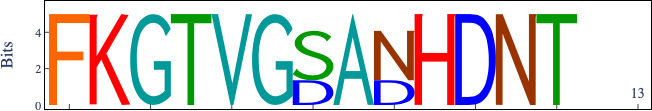	3	8	3	3	2	0
VR7-7 (13)	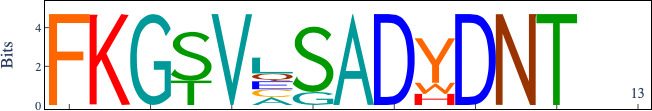	5	13	0	3	10	0
VR7-8 (13)	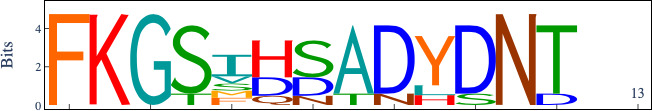	4	5	0	1	2	2
Loop 8							
VR8-1 (16)	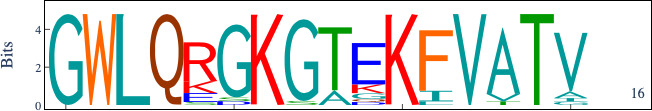	19	361	0	0	7	354
VR8-2 (16)	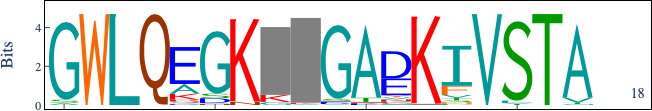	73	5,333	1,724	1,217	2,345	47

^*a*^ aa: amino acid.

Based on phylogenetic analyses and motif identification, alleles from each VR were grouped into representative families (Table 2). VR3, VR5, and VR6 were the most diverse, with VR3 including 7, VR5 9, and VR6 10 peptide families. Several VR families were more frequently associated with P.IA or P.IB. For example, 376 (376/402, 93%) P.IA alleles encoded VR1-2, with only 1 P.IB allele specifying this VR family. By contrast, VR1-4 (2,054/5,304, 39%) and VR1-5 (3,249/5,304, 62%) were more frequently associated with P.IB. VR2-1 (376/402, 93%) was almost exclusively encoded by P.IA alleles, with the majority of P.IB alleles specifying VR2-2 (5,301/5,302, 99%).

VR3-1 was exclusively associated with P.IA, with all of the remaining VR3 families distributed across all 3 P.IB clusters. VR 3-2 was the most frequent VR3 family encoded by P.IB alleles (2,600/5,302, 49%). All P.IA alleles encoded VR5-1 with the most prevalent P.IB VR5 family, VR5-5 (3,238/5,304, 61%) followed by VR5-4 (1,683/5,304, 32%). Similar trends were observed for VR6 and VR7 (Table 2).

Few VR families were shared between P.IA and P.IB, indicating that HGT between PorB classes was rare. Nonetheless, the shorter peptide VR5-1, characteristic of P.IA, was specified by 3 P.IB alleles (NEIS2020_5641, 5499, and 10113) harbored by three isolates originating from the USA (*n* = 2) and Australia (*n* = 1), dating from 2017 to 2019 and belonging to three distinct LINcode lineages indicative of spontaneous, random HGT events between PorB classes that did not lead to expansion. Two of these alleles also encoded VR4-2 peptides, more commonly found in P.IA. In addition, several P.IA alleles possessed VR peptide families more commonly found in P.IB. This included VR1-4, encoded in 5 P.IA alleles harbored by isolates originating from Japan (*n* = 1), Sweden (*n* = 2), and Australia (*n* = 2), dating from 1997 and 2017. These isolates belonged to distinct LINcode lineages and possessed rare PorB designations.

Little to no association was observed between VR families and P.IB clusters, the exceptions being VR3, where VR3-4 was predominantly found in cluster 1 P.IB (568/698, 81%); VR3-5 in cluster 3 (657/1083, 61%); and VR3-3 also in cluster 3 (441/709, 62%) (Table 2). VR3-3, VR3-4, VR3-5, and VR3-6 families consisted of peptide alleles all containing amino acid substitutions known to confer antimicrobial resistance at residues 120 and 121 including G120- > K, N, R, Q, E, and A121- > D, N, G. Finally, the same VR families were identified in isolates dating from the 1940s through to 2022 consistent with VRs that are continually circulating in the gonococcal population (Fig. S2 and S3).

The majority of the Kenyan P.IA alleles encoded VR1-2 (*n* = 11) peptides. Four P.IA alleles, however, encoded the rarer VR2-3 family, which was found in only 17 P.IA alleles harbored by 38 isolates across the whole data set (38/22,227, 0.2%), 14 (37%) of which were from Africa, the remaining isolates originating from Oceania (10/38, 26%), Europe (9/38, 24%), Asia (3/38, 8%), and North America (2/38, 5%) and dating from 1968 to 2020.

### PorB association rule mining

**Fig 2:**
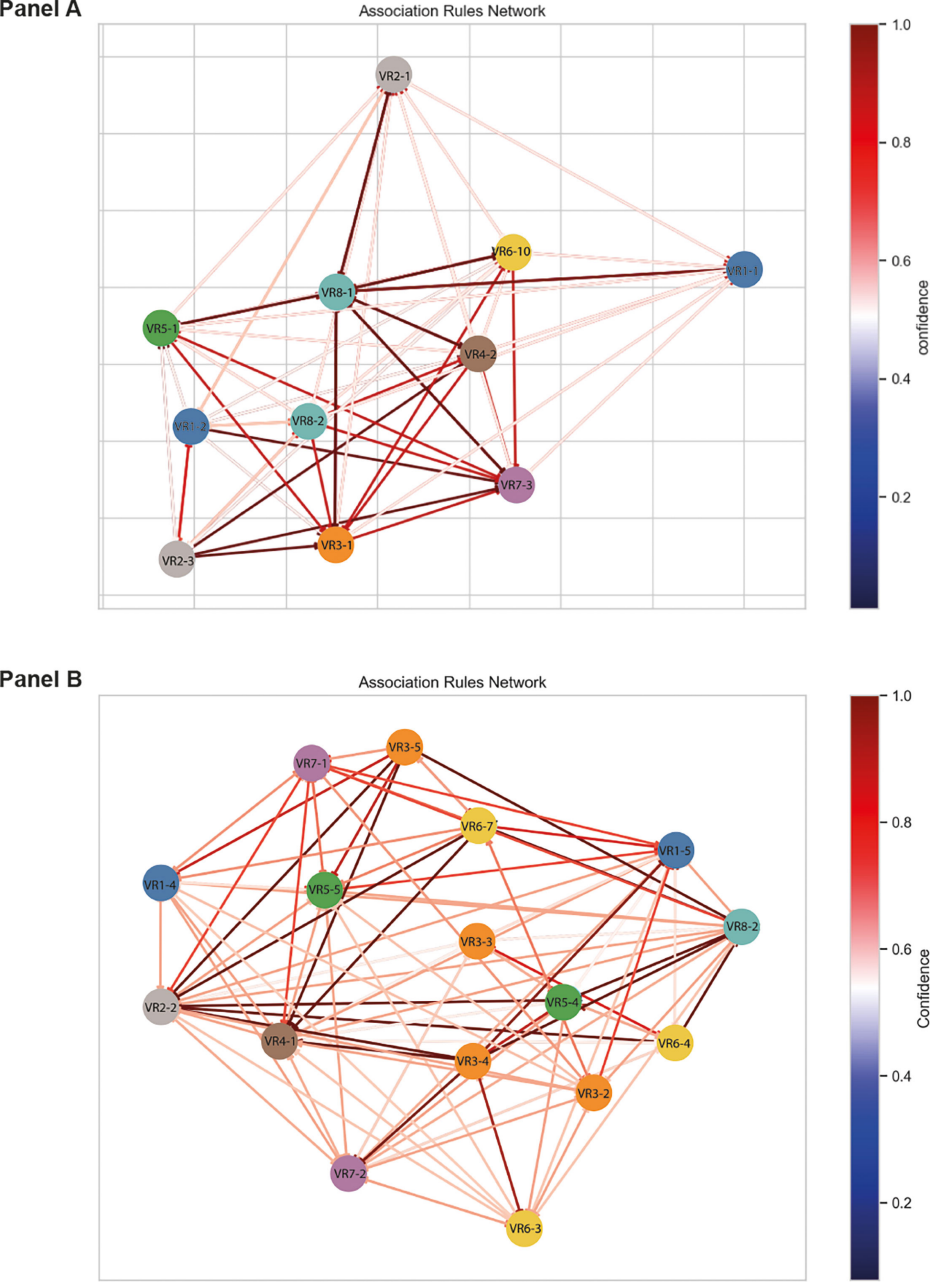
P.IA and P.IB VR networks. VR associations were visualized using the network module in the Python package pyvis.network with nodes colored by VR family and lines denoting the association of antecedent and consequent. Lines were also colored by the confidence values obtained thereby highlighting stronger VR associations. Panel A depicts VR networks obtained for P.IA. The importance of VR3-1, VR5-1, VR6-10, and VR7-3 can be observed. VR1-2 is set apart and has strong links to all other VRs indicated by the darker red lines. (B) Association rule network for P.IB. The distinction between VR1-4 and VR1-5 is visible with preferences between certain VR types noticeable, VR1-4 and VR3-5, for example**,** or VR3-5 and VR5-5, VR3-4, and VR6-3.

Association rule mining identified VR1-2, VR2-1, VR3-1, VR6-10, and VR7-3 as the most frequent P.IA VR families (Fig. S4A) with VR1-4, VR1-5, VR3-3, VR5-5, VR5-4, VR6-7, VR6-3, and VR7-2 and VR7-1 the most prevalent P.IB VR families (Fig. S4B). The association of P.IA VR families VR1-1-VR2-3-VR5-1-VR6-10-VR7-3-VR8-2 had confidence values of 0.538 with a lift of 41.88, indicative of strong associations among these VR variants (Fig. S5A). This association also had zhangs metric values of 1, corresponding to a strong association (17). This included VR families that are the most frequent in P.IA, consistent with the majority of P.IA isolates possessing the designation: PIA:2:1:1:10:3 (2,100/2184, 96%), with network analyses also demonstrating strong links between these VR families (Fig. 2A).

The most frequent P.IB VRs possessed by the gonococci examined here were as follows: VR1-5 (13,306/20,036, 66%), VR1-4 (6,726/20,036, 34%), VR2-2 (20,028/20,036, 99%), VR3-2 (9,895/20,036, 49%), VR4-1 (20,025/20,036, 99%), VR5-5 (12,039/20,036, 60%), VR5-4 (6,780/20,036, 34%), VR6-7 (8,779/20,036, 44%), VR6-3 (7,961/20,036, 40%), VR7-2 (11,323/20,026, 57%), VR7-1 (7,723/20,036, 37%), and VR8-2 (19,994/20,026, 99%) (Fig. S4B). VR1-4- or VR1-5-directed associations were observed. For example, VR3-5 (3,097/3,934, 79%), VR3-6 (357/422, 85%), and VR5-2 (544/649, 84%) were more frequently associated with VR1-4. Whereas VR3-3 (2,371/2,896, 82%), VR3-4 (2,700/2,896, 93%), VR3-2 (7,325/9,897, 74%), VR5-4 (5,988/6,777, 88%), and VR6-4 (2,224/2,515, 88%) were more frequently associated with VR 1-5. Similarly, VR7-1 or VR7-2 associations were observed. VR7-1 was more frequently associated with VR3-5 (2,920/3,492, 84%), VR6-2 (307/368, 84%), and VR6-7 (7,285/8073, 90%), while VR7-2 was more frequently found in combination with VR3-3 (2,676/2,768, 97%), VR3-4 (2,770/2,851, 97%), VR6-3 (7,817/7,849, 99%), and VR6-4 (2,400/2,408, 99%). These associations had corresponding lift, confidence, and zhangs metric values ≥1 (Fig. S5B).

The dichotomy between VR1-4, VR1-5, VR7-1, and VR7-2 and their corresponding VR associations was evident in the network analysis, which revealed a separation between VR1-4, VR1-5, VR7-1, and VR7-2 (Fig. 2B). VR1-4 and VR1-5 share 79% amino acid sequence identity with motif analyses identifying amino acid differences at the peptide terminal (Table 2). A noticeable amino acid difference between VR7-1 and VR7-2 was observed, consisting of a histidine residue in VR7-1 at site 6, while this was aspartic acid in VR7-2 (Table 2). These data indicate that VR1 and VR7 influence P.IB variability and the combination of subsequent VR families present, likely as a result of epistatic, structural, functional, and immunological constraints.

### Analyses of anti-PorB IgG responses following vaccination and natural infection

PorB protein microarray data were obtained from the study by Stejskal et al. (18). These microarrays contained eight diverse PorB subtypes, which were used to assess anti-PorB IgG responses elicited in sera obtained from participants vaccinated with 4CMenB or sera obtained during and after infection (clinicaltrials.gov identification: NCT 04297436). The 8 PorB subtypes on the microarray consisted of PIA:2:1:1:10:3 (NEIS2020_699); PIB:4:2:5:3:2 (NEIS2020_200); PIB:4:2:2:3:2 (NEIS2020_1103); PIB:4:5:5:7:1 (NEIS2020_544 & 1140); PIB:5:2:5:7:1 (NEIS2020_537); PIB:5:2:5:3:2 (NEIS2020_513); and PIB:5:4:4:3:2 (NEIS2020_517).

**Fig 3:**
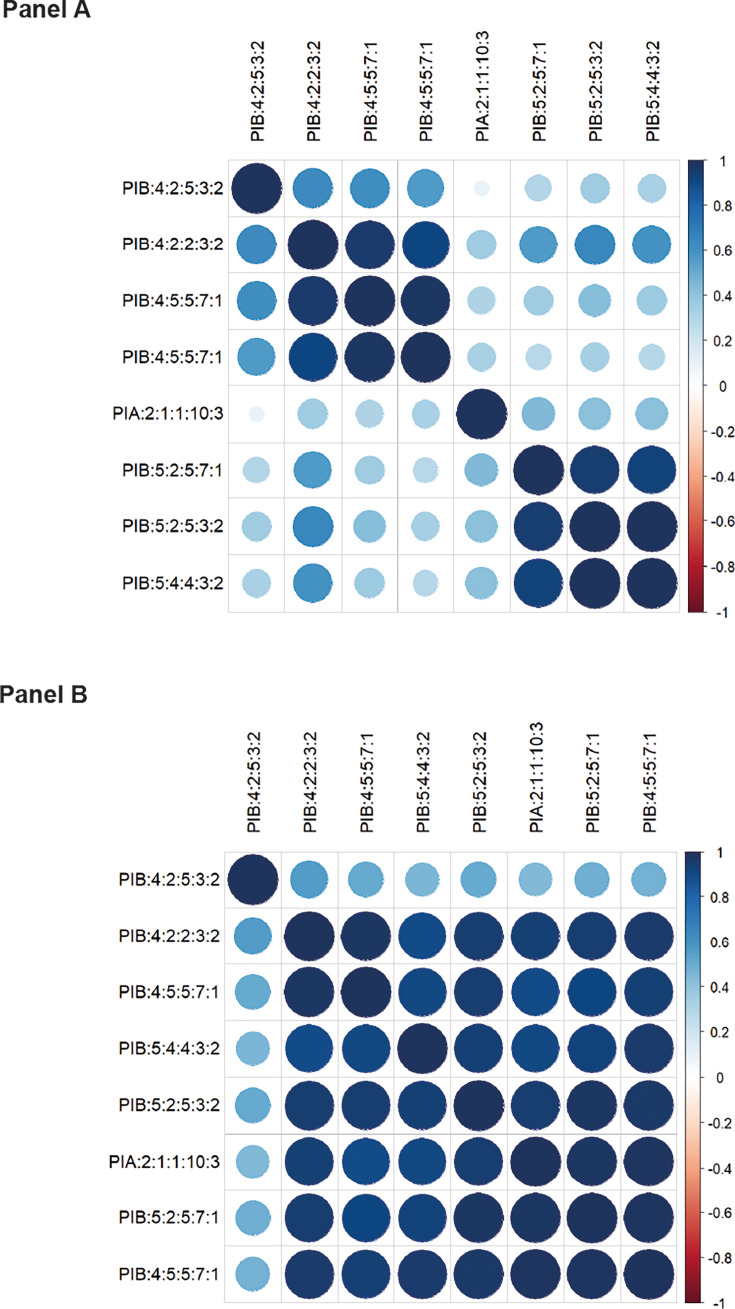
Correlations in IgG reactivities against PorB variants. Pearson correlations in IgG responses from antigen microarray data between different PorB variants are depicted, with the size and color of each correlation shown according to correlation coefficient value, from −1 to +. Each variant is listed by VR type; clustering of similar responses was carried out using the “complete” method using a dedicated script in R. (A) Human Kenya 4CMenB vaccine trial; all data from the t0, t10, and t24 timepoints. (B) Gonorrhea patient cohort; all data from the “Before,” “During,” and “After” timepoints (18).

Microarray analyses on sera following vaccination demonstrated skewed anti-PorB IgG VR responses directed toward distinct VR1-4 or VR1-5 subtypes, indicative of structured, strain-specific antibody responses (Fig. 3A). These data suggest that VR1 elicits strong IgG responses and are consistent with the observed split in network analyses between VR1-4 and VR1-5. Weaker, cross-reactive IgG correlations were observed between VR5:2:5:3:2; PIB:5:4:4:3:2 and PIB:4:2:2:3:2. Although these are different subtypes, they share the same VR6 and VR7 families (VR6-3 and VR7-2) corresponding with the weaker correlation observed and consistent with the cross-reactive immunity found in individuals infected with diverse *N. gonorrhoeae* isolates that share PorB VRs. This was further demonstrated with convalescent sera (Fig. 3B), where no evidence of correlation between PorB subtypes was observed. The prevalence of anti-PorB antibodies in human sera had been noted previously (18), with analyses demonstrating here that infection with one gonococcus will provide partial protection against infection by another genetically diverse gonococcus, should they share PorB VR subtypes (19). The presence of measurable quantities of anti-PorB antibodies suggests that they could play a role in immune selection pressure.

The meningococcal vaccine, 4CMenB, is composed of three recombinant meningococcal proteins: fHbp, neisserial heparin binding protein antigen (NHBA), and neisserial adhesin A (NadA) together with the outer membrane vesicle (OMV) from the New Zealand meningococcal strain MeNZB, NZ98/254 (20). This strain, and therefore the OMV, includes a PorB allele that shares 79%–80% amino acid sequence identity with PIB alleles and 87% sequence identity with PIA. Alignment of consensus amino acid sequences from P.IB originating from clusters 1, 2, and 3, and P.IA revealed that VR1 and VR7 in particular were more homologous with the corresponding VR1 and VR7 in MeNZB than the other variable regions implicated in modulating the immune response (Fig. S6).

### PorB VR designation

**Fig 4:**
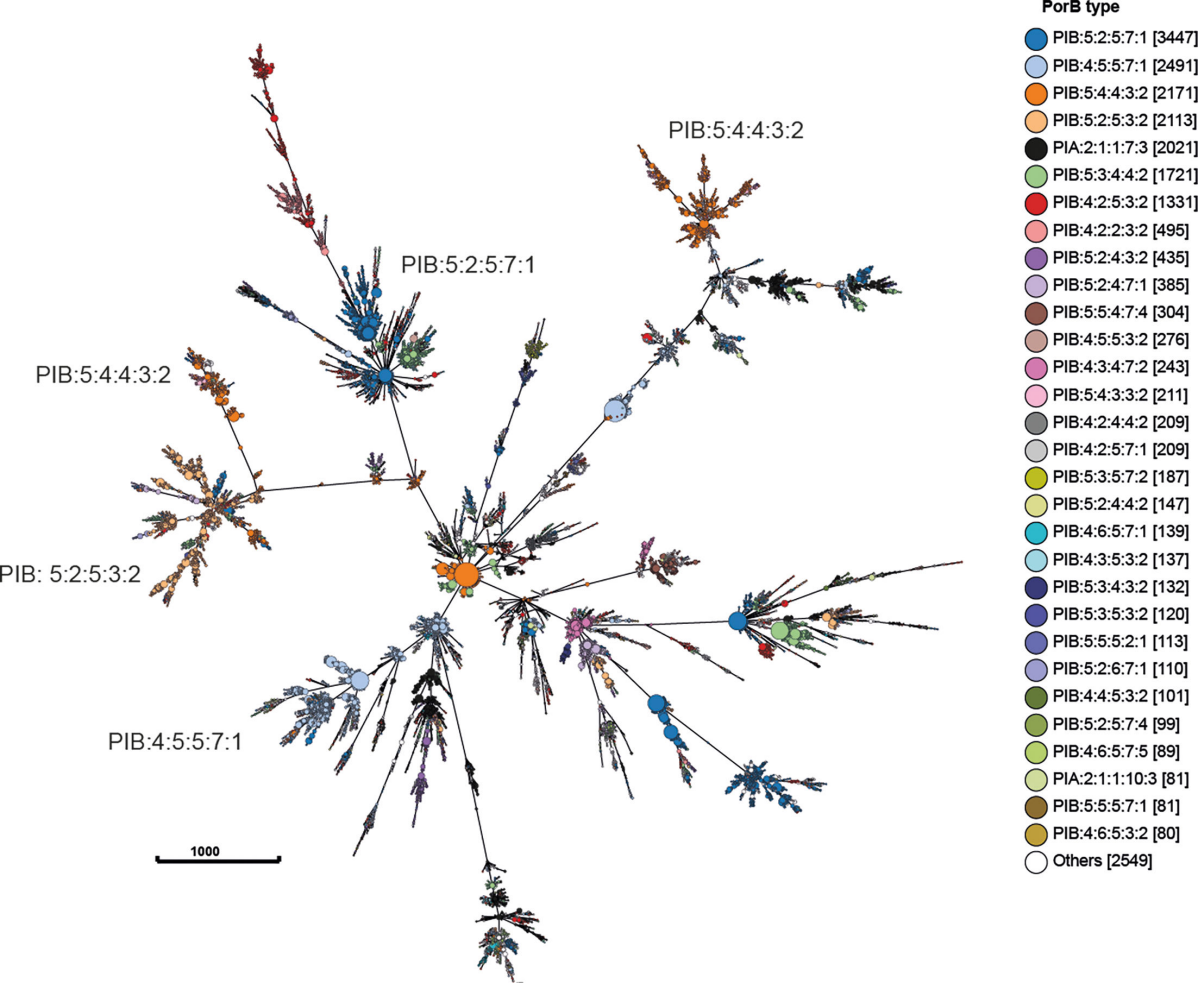
PorB variant distribution in 22,227 gonococci. A minimum spanning tree was generated using the software, GrapeTree, and the *N. gonorrhoeae* cgMLST v2.0 scheme (21). This clusters isolates sharing allelic profiles in the core genome. Each node represents a gonococcal strain with numbers in brackets indicating the number of isolates belonging to that cluster. The more frequent PorB subtypes are indicated in the tree with associations, with core genome observed.

All gonococci deposited in PubMLST possess PorB subtypes that are searchable within the web interface. A total of 308 PorB subtypes were found in the 5,706 *porB* alleles, of which 113 were found once only (Table S1). PIB:5:2:5:7:1 was the most frequent subtype possessed by isolates in this data set (3,447/22,227, 16%) (Table 3; Fig. 4). Although this subtype was found in isolates belonging to 48 LINcode lineages, associations were observed such that, for example, 94% of lineage 1_8_6 isolates harbored subtype PIB:5:2:5:7:1 (692/737), 94% of lineage 1_12_0 (150/159), and 75% of lineage 0_2_15 (986/1309). This subtype was found in 643 P.IB alleles, 634 (634/643, 99%) of which were from cluster 3. Subtype PIB:5:2:5:7:1 was globally distributed, although only found in two isolates from Africa (Kenya and Guinea-Bissau). It was found in isolates dating from 1940 through to 2022, indicative of a subtype that is persisting over time.

**TABLE 3:** Distribution of PorB VR designations per LINcode lineage

PorB VR designation	Lineage (%)												
	0_2_0	0_2_1	0_2_15	0_2_9	0_2_53	0_0_52	0_0_33	0_0_26	0_0_43	1_8_5	1_8_6	1_1_2	1_1_17
PIB:5:2:5:7:1	249 (7)	330 (7)	986 (75)	21 (2)	5 (0.8)	82 (9)	81 (9)	21 (2)	8 (1)	0	692 (94)	378 (57)	19 (3)
PIB:4:5:5:7:1	492 (15)	32 (0.7)	9 (0.7)	74 (8)	17 (3)	679 (71)	42 (5)	1 (0.1)	563 (75)	0	0	39 (6)	1 (0.2)
PIB:5:4:4:3:2	1196 (35)	862 (20)	1 (0.1)	3 (0.3)	5 (0.8)	37 (4)	16 (1.8)	16 (1.8)	1 (0.1)	0	0	0	4 (0.6)
PIB:5:2:5:3:2	17 (0.5)	1613 (38)	34 (3)	116 (12)	13 (2)	41 (4)	8 (0.9)	20 (2.3)	6 (1)	0	0	18 (3)	0
PIA:2:1:1:10:3	496 (15)	66 (1.5)	9 (0.7)	56 (6)	3 (0.5)	1 (0.1)	281 (32)	383 (44)	14 (2)	0	1 (0.1)	59 (9)	14 (2.2)
PIB:5:3:4:4:2	198 (6)	391 (9)	17 (1.3)	14 (1.4)	11 (2)	5 (0.5)	40 (5)	32 (4)	23 (3)	0	2 (0.3)	8 (1)	455 (72)
PIB:4:2:5:3:2	63 (2)	67 (2)	42 (3.2)	23 (2)	2 (0.3)	11 (1.2)	48 (5)	22 (3)	8 (1)	128 (23)	0	4 (0.6)	5 (0.8)
PIB:4:2:2:3:2	4 (0.1)	7 (0.2)	26 (2)	0	0	2 (0.2)	6 (0.7)	3 (0.3)	5 (1)	393 (70)	0	2 (0.3)	1 (0.2)
PIB:5:2:4:3:2	17 (0.5)	70 (1.6)	13 (1)	18 (2)	1 (0.2)	0	0	258 (30)	6 (1)	0	1 (0.1)	21 (3)	2 (0.3)
PIB:5:2:4:7:1	3 (0.1)	100 (2.3)	1 (0.1)	207 (22)	0	0	2 (0.2)	1 (0.1)	3 (0.4)	0	0	2 (0.3)	0
PIB:5:5:4:7:4	6 (0.2)	2 (0.04)	0	1 (0.1)	264 (45)	11 (1.2)	2 (0.2)	0	2 (0.3)	0	0	0	0

PIB:4:5:5:7:1 was the second most frequent subtype found in this isolate collection (2,491/22,227, 11%; Fig. 4). It was distributed in 45 different lineages; however, lineage associations were once again identified with LINcode lineages 0_0_12 (351/449, 78%), 0_0_43 (563/747, 75%), and 0_0_52 (679/950, 71%) subtype, PIB:4:5:5:7:1. This subtype was found in 499 P.IB alleles, 382 of which cluster 3 (382/499, 77%) and was associated with isolates from all continents except Africa. Isolates dated from 1987 through to 2021. Subtype PIB:5:4:4:3:2 was found in 2,171 isolates belonging to 15 lineages, although it was more frequently associated with isolates belonging to LINcode lineages 0_2_0 (1196/3376, 35%) and 0_2_1 (862/4269, 20%) with a much sparser distribution in the 13 other lineages (Table 3; Fig. 4). It was found in isolates dating from 2001 to 2020 across all continents, although only once in Africa. It was found in 382 P.IB alleles, all of which cluster 1.

PIB:5:2:5:3:2 was found in 2,113 isolates belonging to 25 LINcode lineages dating from 1996 to 2020. It was predominantly found in isolates belonging to lineage 0_2_1 (1,613/4,269, 38%) of which 1,257 belonged to sub-lineage 0_2_1_0 (1,257/1,613, 78%), consistent with lineage clonality. PIB:5:2:5:3:2 was found in 365 P.IB alleles, 348 of which cluster 2 (95%). PIA:2:1:1:10:3 was the most frequent P.IA subtype possessed by 2,100 isolates (2,100/2184, 94%) and found in 361 P.IA alleles.

## DISCUSSION

The outer membrane protein PorB is a diverse gonococcal antigen (5) with multiple isolates possessing unique alleles. Extensive diversity will, however, conflict with the essential role PorB plays in gonococcal biology, potentially impeding functionality. Mosaicism and intraspecies genetic reassortment events resulting in alternative PorB conformations must therefore be positively selected for or inversely, negatively selected against, to safeguard the essential role PorB plays in gonococcal viability and infection (22). Here, PorB VR typing combined with machine learning tools identified non-random PorB VR interactions, in spite of high levels of genetic exchange, consistent with the presence of functional constraints, selection of specific VR combinations, and linkage disequilibrium. In particular, specific VR1 and VR7 combinations were found toward which distinct anti-PorB IgG responses were directed, indicating that these loops may be important targets of the immune response. The results presented here can therefore guide OMV-vaccine development with formulations consisting of a cocktail of immunodominant PorB VRs.

In the 1980s and 1990s, gonococci were classified into serotypes or serovars and by auxotype. Serotyping used monoclonal antibodies directed at PorB epitopes (then known as Protein I) while auxotyping denoted distinct nutritional “auxotype” profiles, including, for example, proline-requiring or arginine-requiring gonococci (23). Multiple serovars and auxotypes were defined (23, 24), and correlations between auxotype and serovar were observed (25). These studies, undertaken in the pre-genomic era, had therefore already observed discrete gonococcal populations that segregated by metabolic profile in association with PorB serovar and are consistent with our observations here. Metabolic adaptation is known to play a central role in facilitating gonococcal colonization and infection (26) with the presence of discrete PorB subtypes, in combination with distinct core genome lineages, possibly arising as a result of the gonococcal population naturally segregating into separate metabolic types to limit competition for host nutrients and receptors during infection (27). Non-overlapping, multi-locus associations will therefore arise between antigenic, that is, PorB, and metabolic types to minimize competition and maximize fitness (28).

In addition to nutrient acquisition, which as a porin, PorB facilitates, gonococcal persistence will also depend on the ability to evade the host immune response and antimicrobial treatment, with PorB playing a central role in modulating this. Immune evasion is achieved through the binding of a negative regulator of the complement system, C4B binding protein (C4BP) to VR1 (surface-exposed loop 1) in gonococci expressing P.IA variants which suppresses the complement cascade (29) while AMR is facilitated through distinct amino acid substitutions at residues 120 and 121 in VR3 (surface-exposed loop 3). Consistent with this, DNA hybridization experiments using probes targeting PorB surface-exposed loops had observed the persistence of certain PorB VR types, with differences in P.IA VR1, in particular, conferring intermediate to high levels of serum resistance (30–32). These results are in agreement with the identification here of P.IA isolates, including the serum-resistant strain FA19, possessing the same VR1-2 family with little deviation from this. By contrast, serum resistance has been attributed to loops 5 and 7 in P.IB with evidence that, together, both VRs form a C4BP-binding region (29). This supports the observation here of associations between VR5 and VR7 families in P.IB, demonstrating epistatic interactions between VR5 and VR7 that influence the capacity for serum resistance, with beneficial combinations positively selected for. Association rule mining has previously been used in human genetics studies to identify patterns in complex data sets (33, 34), and employed here shows how it can be used to elucidate patterns conferring selective advantages in the gonococcus.

The dominant role of VR1 in influencing subsequent VR permutations and the immune response was further observed through network visualization of association rules and protein microarray analyses. Network analyses identified a dichotomy between VR1-4 and VR1-5 in P.IB with interactions favoring some VR families over others. The central role of VR1-4 or VR1-5 was further observed through the identification of structured anti-PorB IgG responses in sera from individuals vaccinated with 4CMenB. Serovar-specific immunity has been described previously, with the results presented here indicating that VR1-4 or VR1-5 may influence immune responses (19). Comparison of consensus P.IB amino acid sequences with the PorB from the meningococcal New Zealand strain MeNZB, N98/254, revealed that loops 1 and 7 in particular shared sequence homology and are consistent with the observed skewed IgG responses using protein microarrays. These data indicate that immune responses target distinct PorB regions and that epitopes belonging to the same VR family but, possessed by different PorB subtypes, will generate cross-reactive responses. Thus, the presence of PorB VR types, which are distributed in multiple genome lineages, suggests that cross-protective immunity will occur, whereby infection with one gonococcus from a genetically diverse genome lineage will provide partial protection against infection by another genetically diverse gonococcus. This phenomenon, described as original antigenic sin (OAS) or imprinting (35), would especially occur should either gonococcus possess the same PorB VR type or share VR families. Previous work had identified the existence of IgG responses pre-gonorrhea exposure, indicating that other Neisseria species, including *N. meningitidis*, will also generate cross-reactive responses (36). Further comparison of the sequence similarity between PorB VRs from *N. meningitidis* and *N. gonorrhoeae* will be essential to interpret antibody responses to vaccine formulations using *N. meningitidis* OMVs. The increasing incidence of urogenital infections caused by *N. meningitidis* provides opportunities for further cross-reactive immune responses and highlights this as an important area for future investigation (37).

Results presented here indicate that a balance between diversity and function is in place to preserve fitness, with epistasis between VRs and amino acid residues influencing possible VR permutations and, therefore, PorB diversity. Similar interactions may occur in other gonococcal antigens: this study demonstrates the feasibility of harnessing machine learning tools to investigate such interactions in association with genetic lineage at a population level. The discovery of rarer PorB VR subtypes in African data highlights, however, the need for an improved representation of gonococci from low- and middle-income countries, which will be important when devising vaccine formulations containing VR peptides and in ensuring representativeness across the gonococcal population.

## MATERIALS AND METHODS

### Data set

PorB is defined as NEIS2020 in the Neisseria PubMLST database (https://pubmlst.org/organisms/neisseria-spp), from which a total of 5,706 *N*. *gonorrhoeae porB* alleles were extracted (retrieved 6 November 2024). The *N. gonorrhoeae* isolate collection consists of 22,227 publicly available records originating from Europe (*n* = 12,403), North America (4,227), Oceania (3,192), Asia (1,675), Africa (381), and South America (347). Two isolates did not have a country of origin listed. The collection dated from 1940 to 2022, with the largest proportion dating from 2017 (Table S1). Using the tool GrapeTree (21), a minimum spanning tree was generated for all 22,227 *N*. *gonorrhoeae* isolates by comparing allelic profiles in the *N. gonorrhoeae* cgMLST v2.0, with version 2 developed for use with the Life Identification Number (LIN code) system for gonococcal lineage designation (38, 39). LIN codes are a bacterial barcoding system conveying lineage information in the form of hierarchical clustering at sequential thresholds of allelic mismatch (39). The leftmost numbers of the barcode represent the highest thresholds of allelic mismatch and correspond with super-lineage, lineage, and sublineage designations. LIN code provides a stable, portable nomenclature for *N. gonorrhoeae* and has been implemented in PubMLST. The resulting minimum spanning tree was annotated by PorB class, PorB subtype, and LIN code lineage.

### NEIS2020 (*porB*) genetic analyses

The *porB* locus of *N. gonorrhoeae* is assigned to one of two homology groups based on immunological and structural similarity: *porB1a*, encoding P.IA, and *porB1b*, encoding P.IB (40). Each gonococcal *porB* allele is manually curated in PubMLST and annotated with its PorB class. To do this, BLAST searches of *porB* are undertaken for each genome and, following alignment, the homology status of the resulting *porB* nucleotide sequence is determined, annotated, and the new allele is defined. The *porB1a* alleles are identified based on these having a sequence length ranging from 972 to 996 nucleotides, while *porB1b* alleles vary from 1,014 to 1,077 nucleotides.

Global, progressive, multiple sequence alignments of *porB* nucleotide sequences were undertaken using the alignment tool, MUSCLE (12). Alignments were translated into their deduced amino acid sequences from which a neighbor-joining tree with 100 bootstraps was generated. The resulting tree was edited and annotated using iTOL (13). The Python package, Cogent3 (14), was employed to determine pairwise distances using the (JC69) substitution model in all *porB* alleles (15). JC69 values were then visualized through violin plots generated using the seaborn Python package (41).

### PorB loop annotation and typing

Structurally, PorB exhibits 8 surface-exposed loops, known as variable regions (VR), and all 8 VRs were individually defined in PubMLST and organized into a PorB VR scheme (Table 4). To do this, using published PorB loop descriptions (11), peptide sequences for each VR were defined with, for example, the amino acid sequences: n-TSRSVAHHGAQADRVKTATEIA-c (P.IA) and n-TYRSVEHTKGKVSKVETGSEIA-c (P.IB) defined for loop 1, VR1 (Table 4). WGS were then annotated for each VR, identifying new alleles and resulting in VR allelic profiles for each isolate record (Table S1).

**TABLE 4:** Representative PorB amino acid loop sequences used to seed the database^*a*^

Loop	PIA peptide sequence (aa length)	PIB peptide sequence (aa length)	Location in PIA nt (aa sites)	Location in PIB nt (aa sites)	Number of peptide alleles
Loop 1	TSRSVAHHGAQADRVKTATEIA (22)	TYRSVEHTKGKVSKVETGSEIA (22)	97–162 (33–54)	97–169 (34–55)	525
Loop 2	AYVSGTDTGWG (11)	ASVAGTNTGWG (11)	244–276 (82–93)	253–285 (83–93)	50
Loop 3	LNNILKDTDGFNPWEGKSYYLGLSNIAQPEERH (33)	LNSPLKNTGANVNAWESGKFTGNVLEISGMAQREHRY (37)	334–435 (113–145)	343–468 (112–149)	558
Loop 4	VPNDNSGKNRSE (12)	APKDNSGSNGE (11)	493–528 (166–175)	526–561 (168–179)	62
Loop 5	RHNYTTEKH (9)	RYGEGTKMEGYAYNIPSLFVEKL (23)	595–621 (198–207)	628–813 (202–225)	1,278
Loop 6	DAKLTWSNDNSH (12)	DAKLYGTWRANSHN (14)	691–726 (230–242)	907–975 (249–264)	355
Loop 7	FKGSVYDADNDNTY (14)	FKGSVHSADYDNT (13)	805–846 (268–281)	1,051–1,096 (288–300)	136
Loop 8	GWLQRGKGTEKFVATV (16)	GWLQEGKGADKIVSTA (16)	910–957 (305–319)	1,165–1,219 (325–340)	98

^*a*^ AA: amino acid; nt: nucleotide.

A PorB subtyping scheme was developed on the basis of shared VR motifs and phylogenetic analyses. To do this, phylogenetic trees were generated for each VR, allowing clusters of related alleles to be identified. VR families representative of each cluster were then generated with motifs identified using seqlogo from the Python library, cogent3 (14).

PorB VR subtypes, adapted from Ling et al. (30), were then applied to each isolate record. This consists of the PorB class (P.IA or P.IB), followed by the following VRs: P.IA: VR1, 2, 3, 6, and 7 (corresponding to loops 1, 2, 3, 6, and 7); P.IB: VR1, 3, 5, 6, and 7 (loops 1, 3, 5, 6, and 7) with the last digit from each of VR family used in the typing scheme.

### PorB association rule mining

Association rule mining was used to identify patterns and relationships between VR families. This works by identifying the VR allelic frequency found and generating association rules that consist of “if peptide VR1-1 is found in isolate A (antecedent), then how likely is peptide VR2-1 also present (consequent).” To do this, the apriori algorithm was employed which uses a bottom-up approach, starting with individual items, gradually building up to more complex relationships (42). The unsupervised apriori algorithm used the MLxtend (machine learning extensions) Python library with data manipulation and visualization performed using pandas, matplotlib, numpy, and seaborn (43–46).

For a VR to be considered frequent, the minimum support threshold of 0.01 was used for PIA, which represents the minimum number of times a VR family needed to be present for it to be considered frequent (i.e., 1% of the total number of isolates). A minimum support threshold of 0.05 (5%) was used for P.IB. VR families that did not meet this threshold were removed from further analyses. A list of all possible VR combinations was then generated, and the number of times each combination appeared was counted.

Metrics commonly used in association rule mining to evaluate the importance of identified associations consist of (i) support; (ii) confidence, and (iii) lift. Support measures how frequently a VR family is present. It is calculated by dividing the number of isolates containing the VR family by the total number of isolates in the data set. High support indicates that a VR family is common while low support indicates it is rare. Confidence measures the strength of an association between 2 VR families. It is calculated as the number of isolates containing both VR families divided by the number of isolates containing only the first. High confidence indicates that the presence of the first VR family (antecedent) is a strong predictor for the presence of the second (consequent). Lift measures the strength of the association between 2 VR families, taking into account the frequency of both families in the data set. It is calculated as the confidence of the association divided by the support of the second family. A lift value greater than 1 indicates that the association between two peptide families is stronger than expected based on the frequency of the individual VR family, suggesting that the association may be important.

The top 50 VR frequencies were visualized using the Python package seaborn. 3D pivot plots depicting the support, confidence, and lift values obtained for each association were generated using the Python packages seaborn and matplotlib. VR associations were also visualized using the network module in the Python package pyvis.network with nodes colored by VR family and arrows denoting the direction of antecedent and consequent. Arrows were also colored by the confidence values obtained, thereby highlighting stronger VR associations.

### PorB immunogenicity analyses with Kenyan plasma samples

Gonococcal protein microarray slides, as described previously (18), were employed to assess IgG responses to PorB. These slides include 91 recombinant gonococcal protein samples, of which there are eight PorB alleles that were chosen based on their frequency in the gonococcal population. These include alleles: NEIS2020_699 (P.IA, PIA:2:1:1:10:3) and the P.IB alleles: 200 (FA1090 PorB, PIB:4:2:5:3:2), 513 (PIB:5:2:5:3:2), 517 (PIB:5:4:4:3:2), 537 (PIB:5:2:5:7:1), 544 (PIB:4:5:5:7:1), 1103 (PIB:4:2:2:3:2), and 1140 (PIB:4:5:5:7:1). Each microarray slide consisted of 16 identical blocks, with each miniarray containing 91 gonococcal protein preparations and 17 control samples printed in five repeats.

PorB immunogenicity probing using the microarray slides was conducted as described previously using plasma samples obtained from (i) Kenyan patients with laboratory-confirmed gonococcal infection (*n* = 47) for which there were matched *N. gonorrhoeae* whole genome sequence data (Table S2) (47) and (ii) participants in the clinical trial (clinicaltrials.gov identification: NCT 04297436) of Bexsero, a licensed *N. meningitidis* vaccine undertaken in Kenya between June 2021 and February 2022 (18) (*n* = 50). The correlation matrix was calculated and displayed in graphical form using the corrplot package (version 0.92), run in R (version 4.4.1). PorB variant data were taken from the study by Stejskal et al. (18).

## SUPPLEMENTAL MATERIAL

Figure S1 mbio.01309-25-s0001.docx MST depicting all isolates labeled by LINcode lineage.

Figure S2 mbio.01309-25-s0002.tif PIA VR temporal distributions.

Figure S3 mbio.01309-25-s0003.tif PIB VR temporal distribution.

Figure S4 mbio.01309-25-s0004.tif VR family frequencies.

Figure S5 mbio.01309-25-s0005.tif 3D plots depicting statistical measures of associations.

Figure S6 mbio.01309-25-s0006.tif Amino acid alignment.

Supplemental Tables mbio.01309-25-s0007.xlsx Tables S1 and S2.

ASM does not own the copyrights to Supplemental Material that may be linked to, or accessed through, an article. The authors have granted ASM a non-exclusive, world-wide license to publish the Supplemental Material files. Please contact the corresponding author directly for reuse.
