# Supplementary material for: Investigating genetic, antigenic, and structural diversity in the Neisseria gonorrhoeae outer membrane protein, PorB: implications for vaccine design
Source: mBio. 2025 Aug 25;16(10):e01309-25. doi: 10.1128/mbio.01309-25 (PMC12509796; doi:10.1128/mbio.01309-25)
Supplement: Figure S1 — MST depicting all isolates labeled by LINcode lineage. [file mbio.01309-25-s0001.docx]

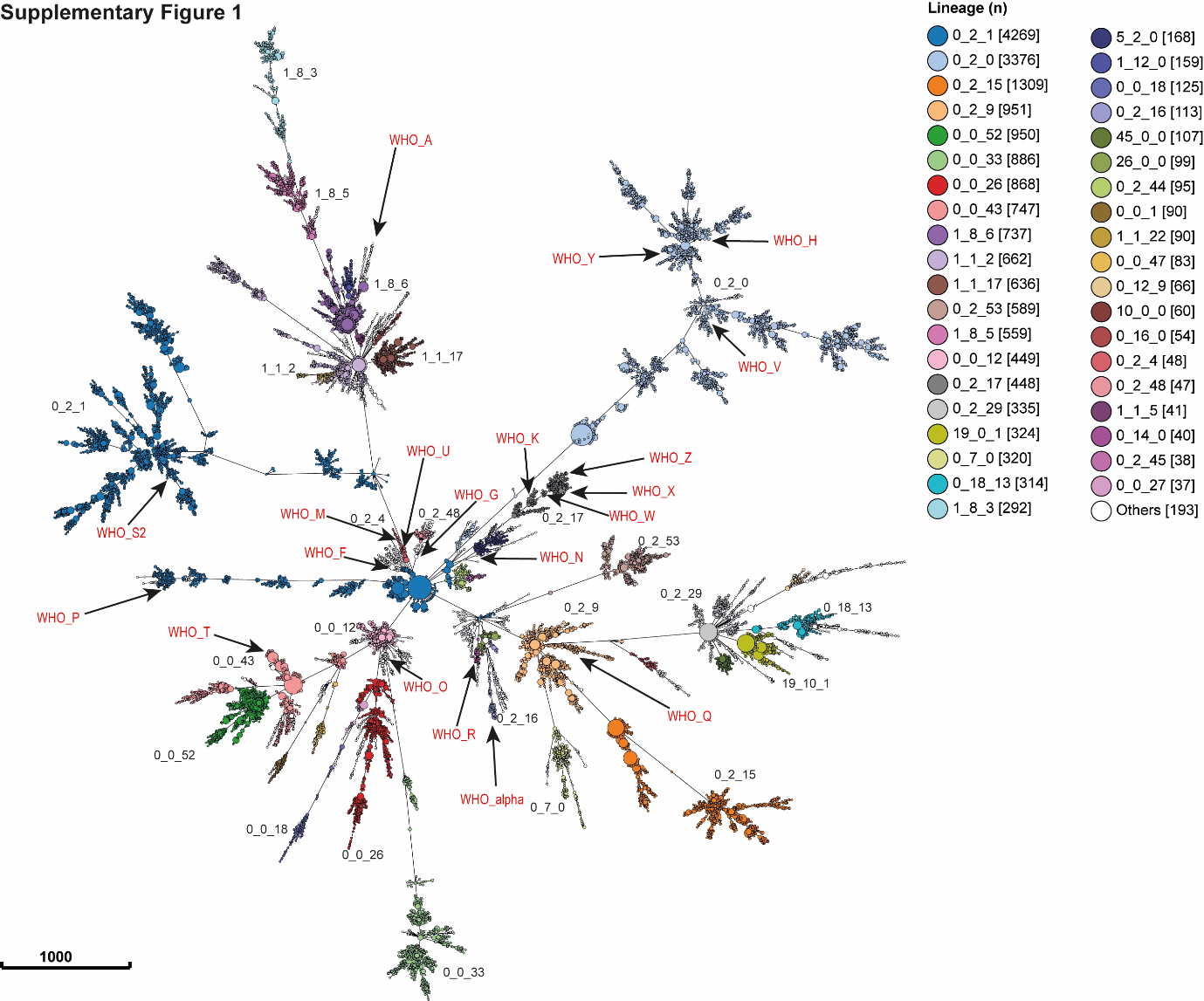


Minimum spanning tree generated using Grapetree and comparing all gonococci using cgMLST v2.0 [34, 47]. Isolates are coloured by LINcode lineage with numbers in brackets indicating the number of isolates belonging to each lineage. The WHO reference isolates are depicted in red with relevant lineages indicated next to each cluster [48].

REFERENCES

34. Unitt, A., et al., *Neisseria gonorrhoeae LIN codes: a Robust, Multi-Resolution Lineage Nomenclature.* bioRxiv, 2025: p. 2025.03.28.646058.

47. Zhou, Z., et al., *GrapeTree: visualization of core genomic relationships among 100,000 bacterial pathogens.* Genome Research, 2018. **28**(9): p. 1395-1404.

48. Unemo, M., et al., *The novel 2024 WHO Neisseria gonorrhoeae reference strains for global quality assurance of laboratory investigations and superseded WHO N. gonorrhoeae reference strains-phenotypic, genetic and reference genome characterization.* Journal of Antimicrobial Chemotherapy, 2024. **79**(8): p. 1885-1899.
