# Supplementary figures and images for: Investigating genetic, antigenic, and structural diversity in the Neisseria gonorrhoeae outer membrane protein, PorB: implications for vaccine design
Source: mBio. 2025 Aug 25;16(10):e01309-25. doi: 10.1128/mbio.01309-25 (PMC12509796; doi:10.1128/mbio.01309-25)

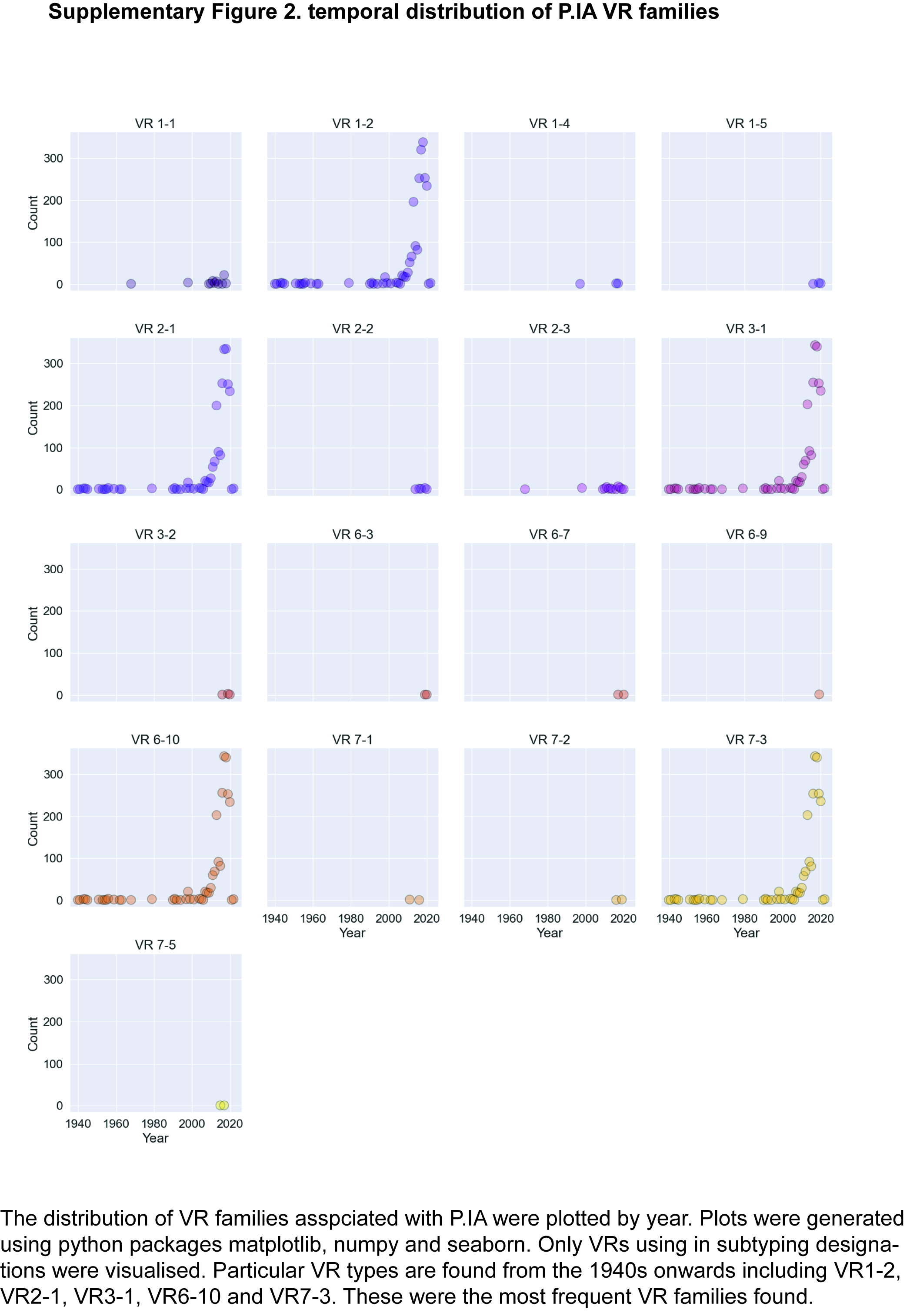

Supplement: Figure S2 — PIA VR temporal distributions. [file mbio.01309-25-s0002.tif]

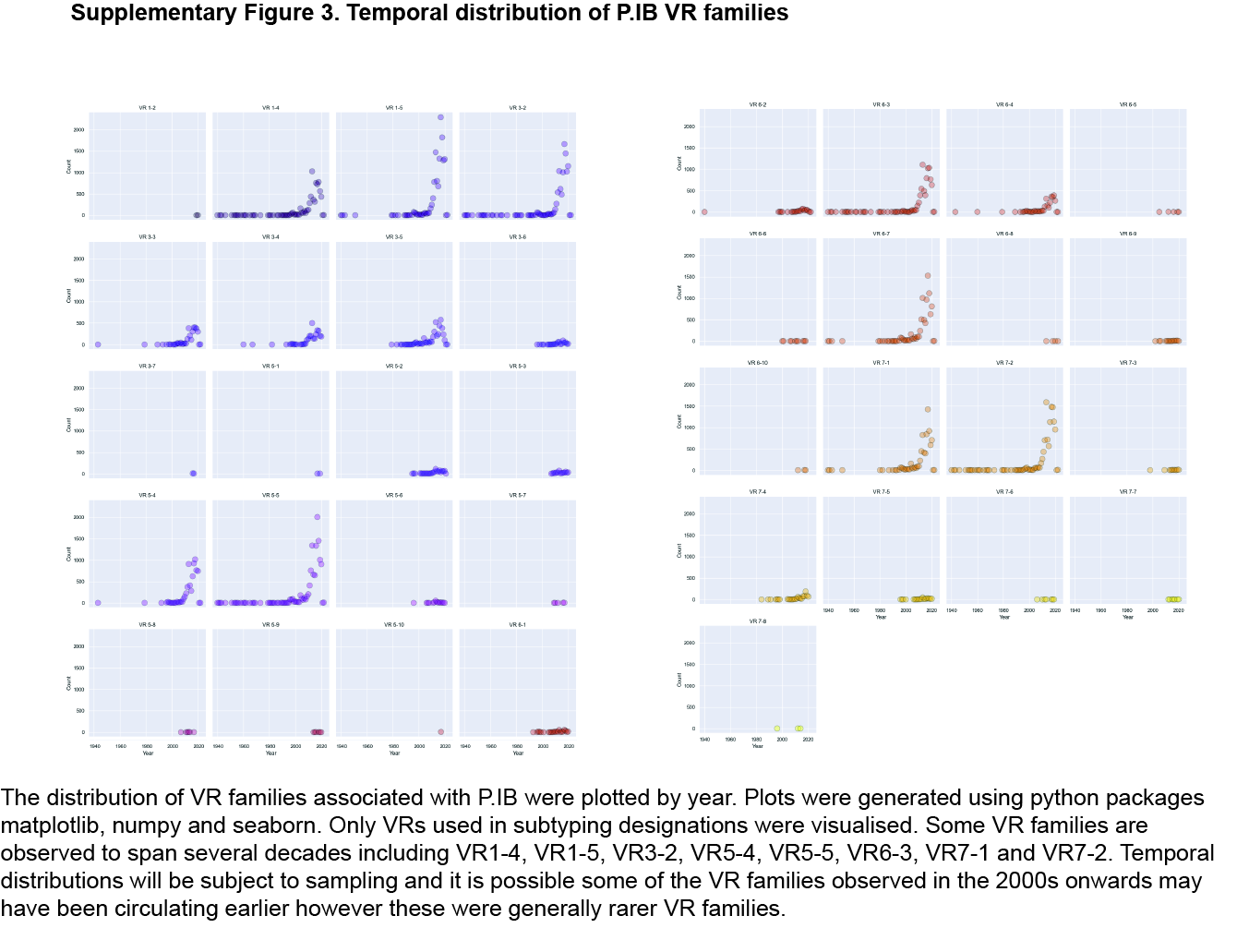

Supplement: Figure S3 — PIB VR temporal distribution. [file mbio.01309-25-s0003.tif]

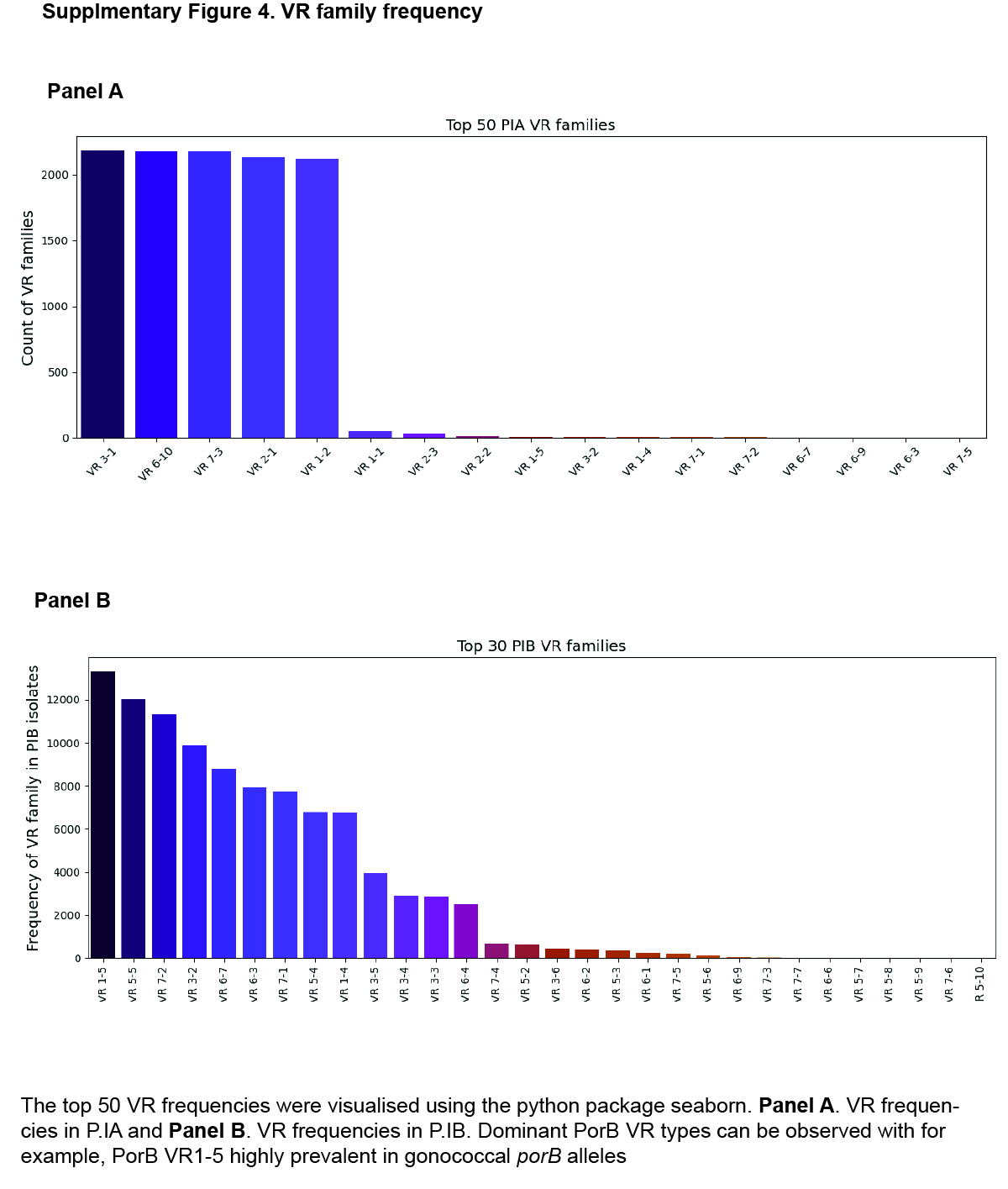

Supplement: Figure S4 — VR family frequencies. [file mbio.01309-25-s0004.tif]

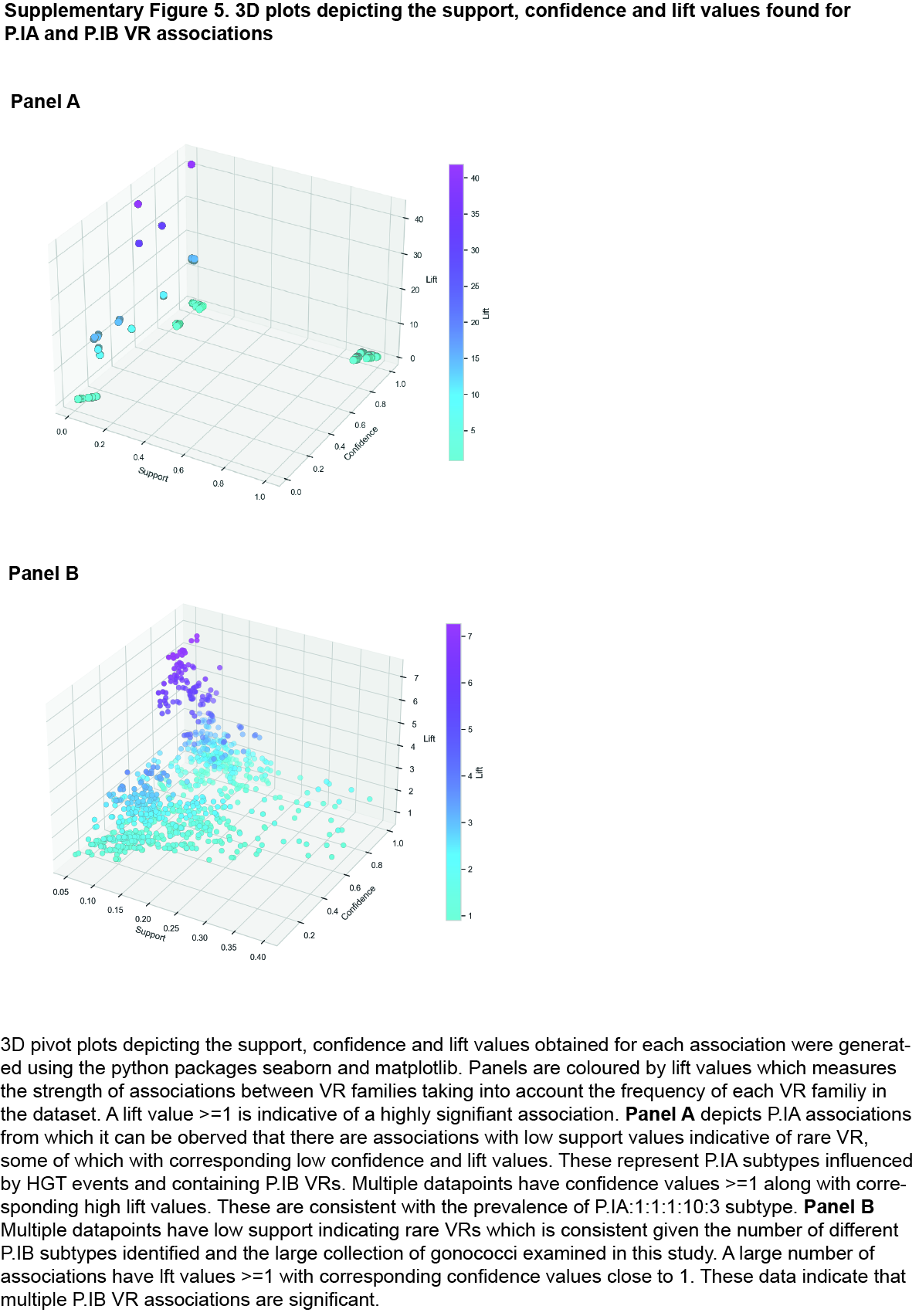

Supplement: Figure S5 — 3D plots depicting statistical measures of associations. [file mbio.01309-25-s0005.tif]

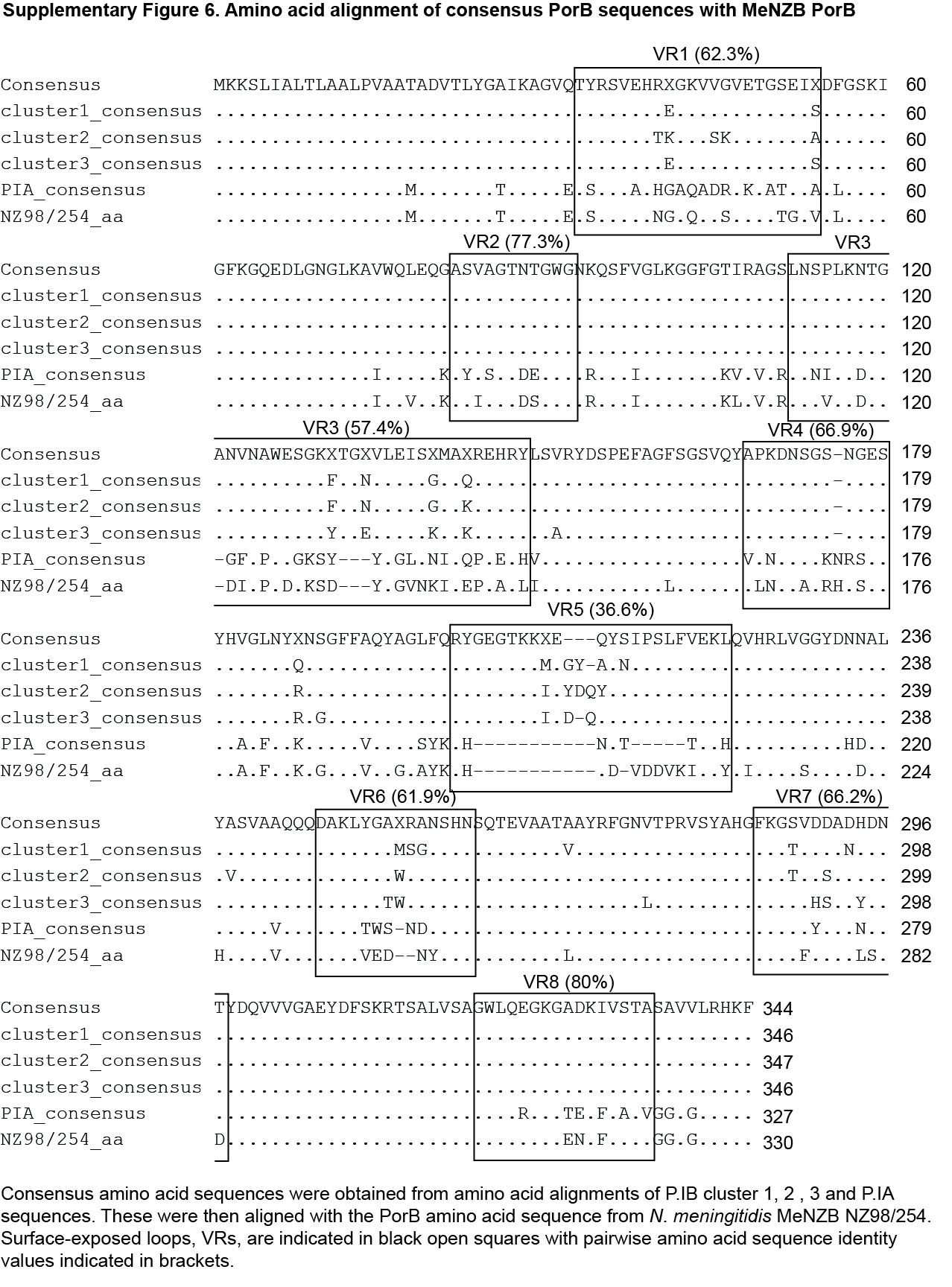

Supplement: Figure S6 — Amino acid alignment. [file mbio.01309-25-s0006.tif]
